# Nasal immune gene expression in response to azelastine and fluticasone propionate combination or monotherapy

**DOI:** 10.1002/iid3.571

**Published:** 2021-11-23

**Authors:** Annabelle M. Watts, Nicholas P. West, Peter K. Smith, Ping Zhang, Allan W. Cripps, Amanda J. Cox

**Affiliations:** ^1^ School of Medical Science Griffith University Southport Queensland Australia; ^2^ Menzies Health Institute of Queensland Griffith University Southport Queensland Australia; ^3^ Queensland Allergy Services Clinic Southport Queensland Australia; ^4^ School of Medicine Griffith University Southport Queensland Australia

**Keywords:** allergic rhinitis, gene expression, mucosa, nasal spray

## Abstract

**Background:**

The combination of the antihistamine azelastine (AZE) with the corticosteroid fluticasone propionate (FP) in a single spray, has been reported to be significantly more effective at reducing allergic rhinitis (AR) symptoms than treatment with either corticosteroid or antihistamine monotherapy. However, the biological basis for enhanced symptom relief is not known. This study aimed to compare gene expression profiles (760 immune genes, performed with the NanoString nCounter) from peripheral blood and nasal brushing/lavage lysate samples in response to nasal spray treatment.

**Methods:**

Moderate/severe persistent dust mite AR sufferers received either AZE (125 μg/spray) nasal spray (*n* = 16), FP (50 μg/spray) nasal spray (*n* = 14) or combination spray AZE/FP (125 μg AZE and 50 μg FP/spray) (*n* = 14) for 7 days, twice daily. Self‐reported symptom questionnaires were completed daily for the study duration. Gene expression analysis (760 immune genes) was performed with the NanoString nCounter on purified RNA from peripheral blood and nasal brushing/lavage lysate samples.

**Results:**

In nasal samples, 206 genes were significantly differentially expressed following FP treatment; 182 genes downregulated (−2.57 to −0.45 Log2 fold change [FC]), 24 genes upregulated (0.49–1.40 Log2 FC). In response to AZE/FP, only 16 genes were significantly differentially expressed; 10 genes downregulated (−1.53 to −0.58 Log2 FC), six genes upregulated (1.07–1.62 Log2 FC). Following AZE treatment only five genes were significantly differentially expressed; one gene downregulated (−1.68 Log2 FC), four genes upregulated (0.59–1.19 Log2 FC). Immune gene changes in peripheral blood samples following treatment were minimal. AR symptoms improved under all treatments, but improvements were less pronounced following AZE treatment.

**Conclusion:**

AZE/FP, FP, and AZE had diverse effects on immune gene expression profiles in nasal mucosa samples. The moderate number of genes modulated by AZE/FP indicates alternative pathways in reducing AR symptoms whilst avoiding extensive local immune suppression.

## Key Highlights

- Antihistamine, steroid and combination nasal sprays elicit distinct gene expression profiles in nasal mucosa samples.
- Modest mucosal gene expression changes were noted for combination therapy compared with a steroid‐only formulation.

## Graphical abstract

Combination nasal spray compared with single active ingredients had diverse effects on immune gene expression profiles in nasal mucosa samples from allergic rhinitis (AR) patients. The moderate number of genes modulated by combination product indicates alternative pathways in reducing AR symptoms whilst avoiding extensive local immune suppression.

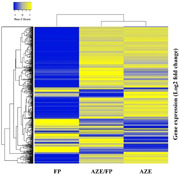

## 1 INTRODUCTION

Allergic rhinitis (AR) is associated with significant global medical and economic burden.^1^, ^2^, ^3^ Intranasal antihistamines and corticosteroids are first‐line treatments for AR management. A combination spray is available for moderate‐to‐severe AR sufferers who require multiple therapies to achieve symptomatic relief.^4^ Clinical trials comparing each active agent (antihistamine or corticosteroid) versus the combination therapy, have demonstrated that the combination spray was more effective than either monotherapy at reducing AR symptoms.^5^, ^6^, ^7^, ^8^, ^9^ As antihistamines and corticosteroids have distinct mechanisms of action, potential additive, or synergistic effects may contribute to the enhanced symptomatic relief observed. Experimental studies examining the mechanism of action behind these enhanced effects are limited. The purpose of this study was to investigate the potential mechanisms through which antihistamine and corticosteroid nasal sprays provide relief from AR symptoms and to determine if combining an antihistamine and corticosteroid provides any synergistic effects on gene expression profiles in the nasal mucosa and blood samples.

## 2 METHODS

### 2.1 Study design

This study was a randomized, double‐blind, three‐armed parallel‐group study where combination therapy was compared against single active ingredients as control groups. Clinical assessments were conducted at the Queensland Allergy Services Clinic (Gold Coast, Australia) and the Clinical Trial Unit at Griffith University (Gold Coast, Australia) from November 2016 to May 2018. Participants attended a screening visit (Day 14) for evaluation of allergen sensitivities and provision of blood samples. Following screening, eligible participants were instructed to complete a 14‐day washout period and cease use of all intranasal and immune modulating medications. Participants were advised to use the following as rescue medication in the event of a considerable symptomatic episode: (1) nasal irrigation with saline solution, (2) oral decongestants, and (3) oral antihistamines. Participants were requested not to take any allergy medications in 48 h before the screening (Day 14) and baseline (Day 0).

At baseline, participants provided nasal lavage/brush and blood samples for gene expression analysis. Participants were randomized using a block randomization method stratified by allergen sensitivity (dust mite only or dust mite and grass allergy) and sex to one of three treatment groups (1) Azep® nasal spray [Mylan Health Pty Ltd], azelastine (AZE) 125 µg/spray, (2) Flixonase® nasal spray [Mylan Health Pty Ltd], fluticasone propionate (FP) 50 µg/spray, or (3) Dymista® nasal spray [Mylan Health Pty Ltd], 125 µg of azelastine, and 50 µg fluticasone propionate/spray (AZE/FP). Participants were instructed to administer the allocated nasal spray (provided in a sealed envelope to maintain blinding) 1 spray per nostril, twice daily, for 7 days. Participants were discouraged from using any allergy medications other than the study medication during the treatment period. Following the treatment period, participants returned for the final visit (Day 7) for the provision of nasal lavage/brushing and blood samples. Participant compliance was assessed based on a self‐report of the number of doses missed and was also estimated by measuring the amount of study medication remaining (by weight) relative to the amount (weight) before dispensing.

This study was approved by the Griffith University Human Research Ethics Committee (Ref:2016/279) and was registered with the Australian and New Zealand Clinical Trial Registry (ACTRN12616001439437) before commencement. All participants provided written and informed consent before participation.

### 2.2 Participant selection: Inclusion and exclusion criteria

Men and women, 18–65 years of age, with a more than 2‐year history of moderate‐severe AR (as defined by the Allergic Rhinitis and Its Impact on Asthma (ARIA) guidelines)^10^ were recruited to the study. Participants were also required to have a Total Nasal Symptom Score (TNSS)^11^ of at least six, a score of at least 50 mm on a Visual Analog Scale (VAS) for overall symptom severity^12^ in the previous 24 h, and a positive allergic response to dust mites determined with a skin prick test and/or serum specific IgE radioallergosorbent test (RAST) (QML Pathology, Murarrie, Queensland, Australia) to *Dermatophagoides pteronyssinus* or *D. farinae*.

Individuals suffering from nonallergic rhinitis; who consumed probiotics in the previous 12 weeks, used oral corticosteroids within the previous 6 months or antibiotics within the previous 30 days; used anti‐inflammatory/immune‐modulating medications; had the existing respiratory disease (including asthma, nasal polyposis, or chronic obstructive pulmonary disorder); had existing immune dysfunction (other than allergies); had recent nasal surgery/trauma that could affect sampling; were ill at time of enrollment; reported hepatic impairment or excessive alcohol consumption^13^; had known hypersensitivity to steroids or antihistamines; or were pregnant at the time of enrolment were excluded from participating.

### 2.3 Symptom assessment

Participants completed the mini Rhinoconjunctivitis Quality of Life Questionnaire (mRQLQ)^14^ at the beginning and end of the intervention and maintained a symptom and medication diary (SMD) daily for the duration of the study. The SMD consisted of three symptom questionnaires: TNSS, total ocular symptom score (TOSS)^15^ and Other Allergic Rhinitis Symptom Score (OARSS), in addition to a VAS for overall symptom severity. The use of allergy and non‐allergy‐related medications were also recorded in the diary.

### 2.4 Sample collection and laboratory analysis

A screening blood sample was collected and the following tests conducted: full blood count, white cell differential, erythrocyte sedimentation rate (ESR), and specific IgE to dust mites (*D. pteronyssinus* and *D. farinae*.) and grass pollen mix (Bermuda, Timothy, Meadow, Johnson, Rye and Paspalum) (QML Pathology). For those individuals who met the inclusion criteria nasal washing and brushing, and blood samples were collected at Day 0 and Day 7 visits as described previously.^16^ Briefly nasal samples included both washing with 100 ml of phosphate‐buffered saline and brushing of the mucosa between the nasal septum and inferior turbinate of each nostril. Recovered washing and brushing material was combined and cellular material concentrated by centrifugation. The cell pellet was directly lyzed using a commercially available lysis buffer (RLT; Qiagen) and the lysate was stored frozen until gene expression analysis. For whole blood samples, RNA was extracted from PAXgene tubes using a Maxwell® RSC automated RNA extraction instrument using the commercially available Maxwell® RSC miRNA Tissue Kit (Promega Corporation).

### 2.5 Gene expression analysis

Immune gene expression analysis of nasal cell lysate and extracted RNA from blood was performed using a commercially available NanoString nCounter PanCancer Immune Profiling panel (NanoString Technologies). This panel contained 40 references (housekeeping) genes and 730 immune genes and was used in combination with the nCounter panel plus probe set which contained an additional 30 immune genes relating to the allergic response and mechanism of action of steroids and antihistamines (760 immune genes in total). Gene expression data underwent imaging quality control and normalization checks before analysis and interpretation of data. Genes that were expressed at counts below 20 in 80% or more samples were excluded from further analysis. Reference (housekeeping) normalization was performed using the GeNorm Algorithm where 20 out of 40 housekeeping genes were used for the nasal lysate samples and 33/40 housekeeping genes were used for the peripheral blood samples.

### 2.6 Statistical analysis

Based on a standard deviation of gene expression intensity of 0.6, an *α* of 0.001, and at least two‐fold difference in gene expression, a sample size of 16 patients per group was estimated to achieve 95% power. Differences in demographic and clinical measures between groups were assessed with a one‐way analysis of variance and a *χ* ^2^ test for categorical variables. Variables were log‐transformed where appropriate to approximate a normal distribution. Change (pre‐post) in symptom severity questionnaires was measured with a paired *t*‐test. Differences in absolute change in symptom severity questionnaires between groups were measured with an analysis of covariance with baseline score as the covariate. Differentially expressed genes were identified using R package Limma,^17^ where moderated *t*‐tests were performed to compare the gene expression levels between groups. The significantly differentially expressed genes (*p* < .05) in each treatment group were assessed for enrichment into Reactome pathways. Statistical significance of all clinical measures differentially expressed genes and pathway enrichment was accepted at *p* < .05.

## 3 RESULTS

### 3.1 Study cohort

**Figure 1:**
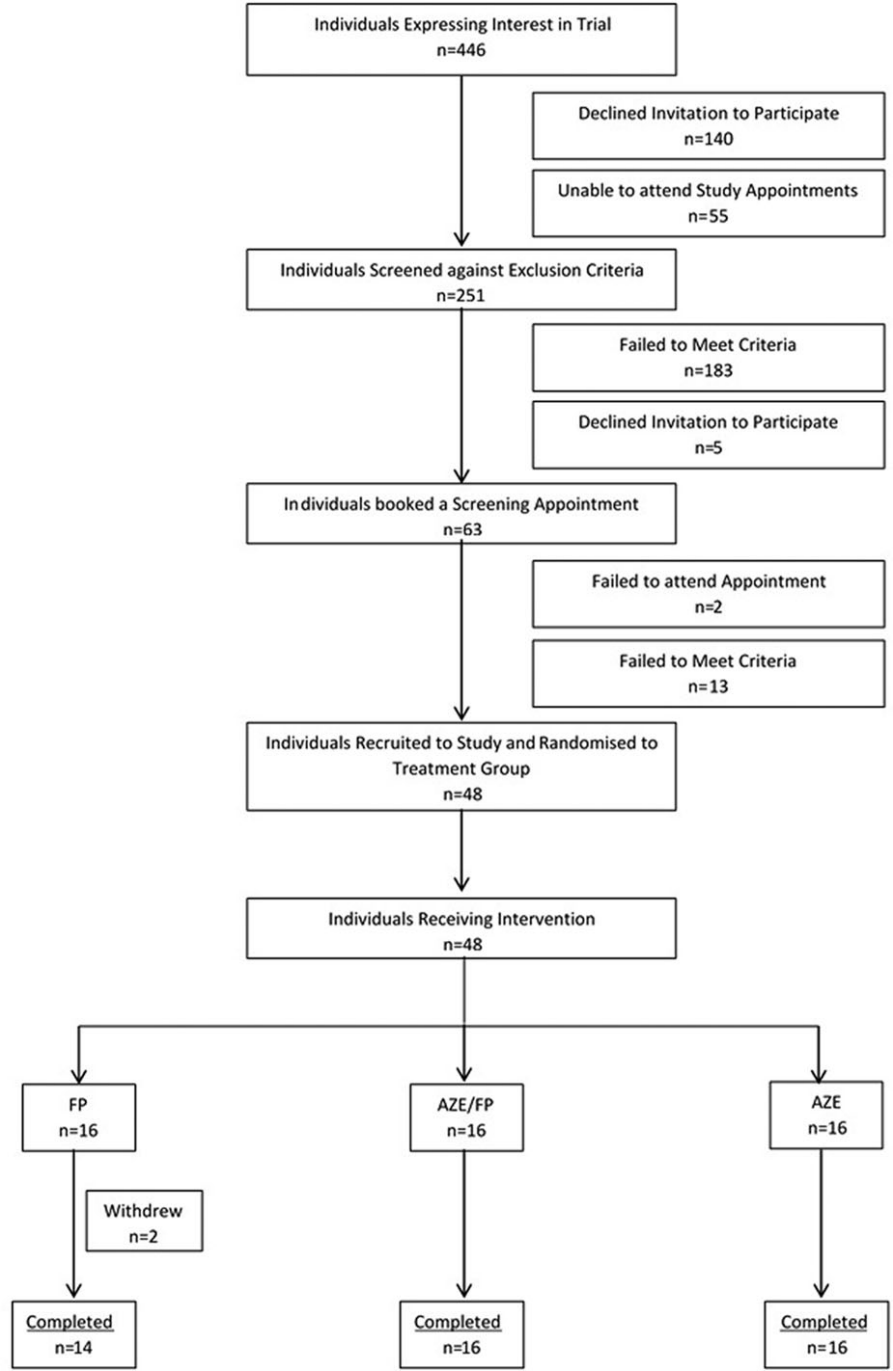
Consort diagram depicting flow and retention of study participants FP, fluticasone propionate “Flixonase®” group; AZE/FP, azelastine/fluticasone propionate “Dymista®” group; and AZE, azelastine “Azep®” group

Forty‐eight participants were randomized to the study. Two participants did not complete the treatment period and were withdrawn (Figure 1). Reasons for withdrawal included the development of the ear infection in one participant and an unexpected adverse event considered unrelated to the intervention in a second participant that prevented compliance with the study protocol. The demographic and baseline characteristics of the study groups based on a per‐protocol analysis are given in Table 1. The groups were matched with the exception of blood eosinophil counts which were significantly different between the FP and AZE groups.

**Table 1:** Baseline demographic and clinical measures for the per‐protocol population

	FP	AZE/FP	AZE	*p* value
*n*	14	16	16	–
Age (years)	37.63 ± 14.60	39.42 ± 10.03	37.26 ± 15.08	.889
Sex F/M (% female)	10/4 (71%)	10/6 (63%)	11/5 (69%)	.864
Height (cm)	169.11 ± 9.35	171.03 ± 10.26	171.94 ± 8.63	.710
Weight (kg)	76.69 ± 15.72	72.16 ± 15.33	73.46 ± 13.20	.694
BMI (kg/m^2^)	26.65 ± 3.95	24.45 ± 3.37	24.78 ± 3.64	.226
Ethnicity (% Caucasian)	78.60%	68.80%	87.50%	.437
Immune measures (Day‐14)				
White cell count (×10^9^/L)	7.18 ± 1.70	6.98 ± 2.09	5.90 ± 1.50	.112
Lymphocytes (×10^9^/L)	2.32 ± 0.80	2.26 ± 0.73	1.98 ± 0.63	.366
Eosinophils (×10^9^/L)	0.53 ± 0.37	0.44 ± 0.30	0.26 ± 0.16	.038
Neutrophils (x10^9^/L)	3.74 ± 1.22	3.68 ± 1.33	3.18 ± 1.00	.366
Basophils (×10^9^/L)	0.06 ± 0.05	0.07 ± 0.03	0.04 ± 0.03	.085
ESR (mm/h)	14.50 ± 13.78	7.94 ± 6.17	7.94 ± 8.27	.267
Allergen sensitivity (Day‐14)				
Co‐allergy to dust mites and pollen (%)	50%	62.50%	62.50%	.731
IgE *Dermatophagoides pteronyssinus* (kU/L)	39.28 ± 40.39	17.12 ± 21.60	11.37 ± 20.34	.088
IgE *D. farinae* (kU/L)	35.11 ± 39.85	13.41 ± 18.31	8.56 ± 17.31	.093
IgE grass pollen mix (kU/L)	2.13 ± 4.29	10.44 ± 25.84	6.20 ± 21.29	.526
IgG4 *D. pteronyssinus* (kU/L)	0.50 + 0.54	0.47 ± 0.42	0.38 ± 0.43	.772
IgG4 *D. farinae* (kU/L)	0.42 ± 0.42	0.39 ± 0.26	0.30 ± 0.38	.617
IgG4 grass pollen mix (kU/L)	0.67 ± 0.35	1.07 ± 0.97	0.80 ± 0.66	.469
Symptom severity (Day 0)				
Total Nasal Symptom Score (0–12 U)	5.93 ± 3.95	4.00 ± 1.86	7.06 ± 3.64	.299
Total Ocular Symptom Score (0–9 U)	3.57 ± 2.44	2.00 ± 2.00	3.19 ± 2.74	.182
mRQLQ Score (0–6 U)	2.90 ± 1.25	2.66 ± 0.79	3.00 ± 1.12	.653
Other allergic rhinitis symptoms (0–12 U)	4.51 ± 3.89	3.07 ± 2.16	5.21 ± 3.47	.865
Visual Analog Scale (0–100 mm)	54.18 ± 33.51	48.16 ± 22.31	60.50 ± 30.10	.485
Medication Usage (Day 14–Day 0)				
Allergy medication use; % of total diary responses (washout period)	0.37 ± 0.29	0.25 ± 0.20	0.40 ± 0.31	.237

Abbreviations: AZE, azelastine “Azep®” group; AZE/FP, azelastine/fluticasone propionate “Dymista ®” group; FP, fluticasone propionate “Flixonase ®” Group; mRQLQ, miniRhinoconjunctivitis quality of life.

Treatments were well tolerated by participants. Adverse events were mild, with minimal impact on daily activities, were consistent with previously reported findings^7^, ^18^ and are presented in Table S1. Self‐reported compliance was 96% ± 6% of total scheduled doses for the FP group, 96% ± 6% for the AZE/FP group, and 99% ± 3% for the AZE group. All participants administered ≥86%. Compliance was verified from weights of returned medications and ranged from 81% (AZE group) to 93% (AZE/FP group) missing less than two doses.

Eight of the participants reported needing to follow the rescue medication strategy during the 7‐day intervention period. For five of these participants (two from the FP group, one from the AZE/FP group, and two from the AZE group) this included use of nasal irrigation only. One participant (FP group) reported use of a nasal decongestant, one participant (FP group) reported use of oral antihistamines, and one participant (AZE group) reported use of eye drops.

### 3.2 Symptom assessment

**Figure 2:**
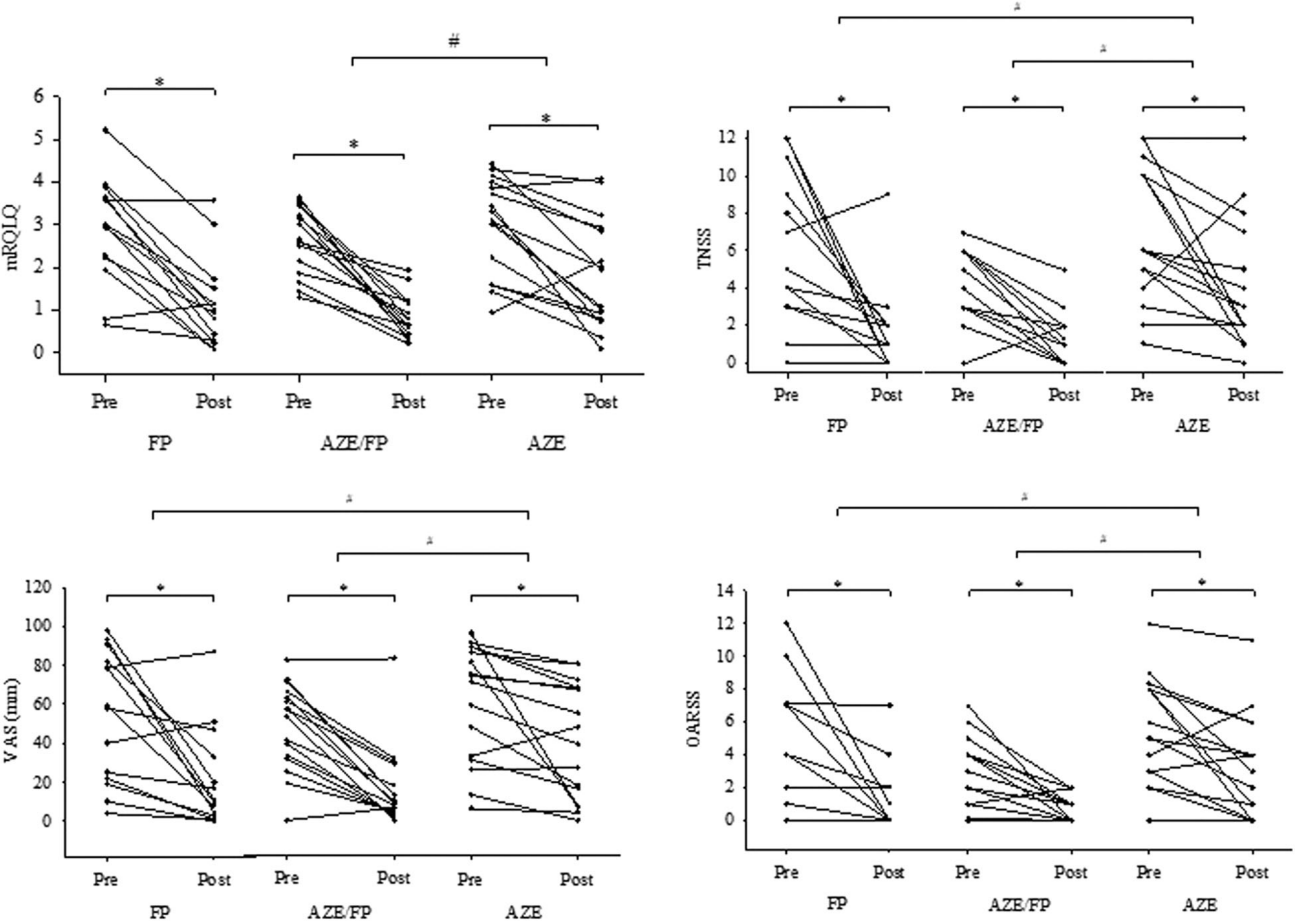
Clinical response to treatment. Dots and lines represent change in symptom scores for each participant from pre‐nasal spray application (Day 0) to post‐nasal spray application (Day 7). The data are shown in the unadjusted values. Asterisk (*) indicates significance at *p* < .05 following nasal spray application. Hash (#) indicates raw change in symptoms scores (pre‐post) was significant between groups (ANCOVA with baseline score as the covariate). ANCOVA, analysis of covariance; AZE, azelastine “Azep®” group; AZE/FP, azelastine/fluticasone propionate “Dymista®” group; FP, fluticasone propionate “Flixonase®” group

All symptom severity measures were significantly improved following treatment in all groups (Table S2). mRQLQ improvement was significantly greater in AZE/FP compared with AZE (*p* = .014) (Figure 2). Both FP (*p* = .013) and AZE/FP (*p* = .016) treatments had a significantly greater effect on TNSS improvement when compared to AZE. Improvement in OARSS was significantly greater in FP (*p* = .029) and AZE/FP (*p* = .044) when compared with the AZE. Reduction in overall symptom severity (based on VAS) was greater in the AZE/FP (*p* = .022) and FP (*p* = .040) when compared with AZE.

### 3.3 Differentially expressed genes

Demographic and clinical characteristics of the subjects who had samples that met the quality control criteria for gene expression studies were not different from the per‐protocol population.

#### 3.3.1 Nasal mucosa

**Figure 3:**
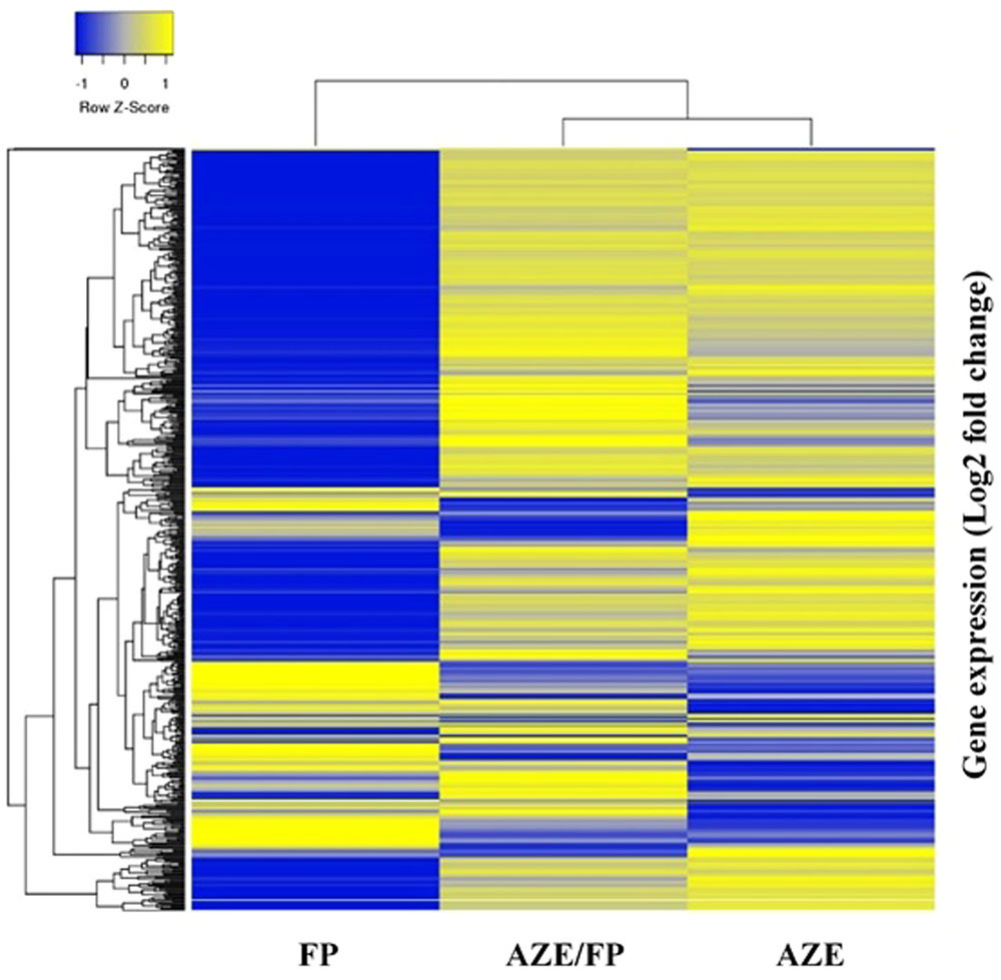
Heat map of Log2 expression all genes in the nasal mucosa samples included in the analysis (*n* = 588) by treatment group. Each row represents a gene and each column represents a sample. The Log2 gene expression counts are represented on a Z scale whereby blue indicates low expression (downregulation) and yellow indicates high expression (upregulation). AZE, azelastine “Azep®” group; AZE/FP, azelastine/fluticasone propionate “Dymista®'”group; FP, fluticasone propionate “Flixonase®” group

Nasal lysate samples from 13 FP, 11 AZE/FP, and 11 AZE participants were available for analysis (Table S3). A total of 588 genes included in the NanoString nCounter panel were expressed above background. FP had a strong downregulatory effect on gene expression, while AZE/FP and AZE had a mostly upregulatory effect on immune gene expression (Figure 3). The top 10 differentially expressed genes for all treatment groups are shown in Table 2.

**Table 2:** Top 10 differentially expressed genes in nasal lysate and blood samples for the three treatment groups

Gene	Log2 fold change	Linear fold change	Lower confidence limit (log2)	Upper confidence limit (log2)	*p* value	*p* adjust
FP Group						
Nasal mucosa samples						
AMICA1	−2.46	0.18	−3.10	−1.83	3.16 × 10^−7^	1.86 × 10^−4^
GZMB	−2.54	0.17	−3.30	−1.78	2.37 × 10^−6^	6.96 × 10^−4^
LTB	−2.41	0.19	−3.20	−1.62	6.83 × 10^−6^	1.03 × 10^−3^
FCER1A	−2.55	0.17	−3.38	−1.71	7.03 × 10^−6^	1.03 × 10^−3^
SOCS1	−2.20	0.22	−3.01	−1.40	2.49 × 10^−5^	2.92 × 10^−3^
PTGDR2	−2.29	0.20	−3.20	−1.38	6.19 × 10^−5^	5.43 × 10^−3^
IL1RL1	−2.36	0.19	−3.33	−1.40	8.39 × 10^−5^	5.43 × 10^−3^
HLA‐DRA	−1.75	0.30	−2.47	−1.03	9.22 × 10^−5^	5.43 × 10^−3^
CXCR3	−2.04	0.24	−2.89	−1.19	1.02 × 10^−4^	5.43 × 10^−3^
CD1C	−1.62	0.33	−2.30	−0.94	1.07 × 10^−4^	5.43 × 10^−3^
Peripheral blood samples						
IL3RA	−0.47	0.72	−0.65	−0.29	4.95 × 10^−5^	2.40 × 10^−2^
IL1B	0.33	1.26	0.15	0.51	1.20 × 10^−^3	2.91 × 10^−1^
MS4A2	−0.38	0.77	−0.62	−0.14	4.45 × 10^−3^	5.14 × 10^−1^
IL5RA	−0.33	0.79	−0.55	−0.12	5.08 × 10^−3^	5.14 × 10^−1^
NFKB1	0.22	1.16	0.07	0.37	6.29 × 10^−3^	5.14 × 10^−1^
RNASE3	−0.47	0.72	−0.79	−0.15	7.12 × 10^−3^	5.14 × 10^−1^
HRH4	−0.29	0.82	−0.49	−0.09	7.43 × 10^−3^	5.14 × 10^−1^
CD24	−0.45	0.73	−0.77	−0.13	9.39 × 10^−3^	5.61 × 10^−1^
SLPI	0.30	1.23	0.08	0.52	1.04 × 10^−2^	5.61 × 10^−1^
NFKB2	0.20	1.15	0.05	0.34	1.22 × 10^−2^	5.91 × 10^−1^
AZE/FP group						
Nasal mucosa samples						
TNFSF10	−0.92	0.53	−1.48	−0.36	3.58 × 10^−3^	9.85 × 10^−1^
NOS2A	−1.53	0.35	−2.57	−0.49	7.43 × 10^−3^	9.85 × 10^−1^
PPBP	1.52	2.87	0.48	2.57	7.66 × 10^−3^	9.85 × 10^−1^
ABCB1	1.23	2.35	0.32	2.14	1.17 × 10^−2^	9.85 × 10^−1^
KIT	−0.98	0.51	−1.76	−0.21	1.66 × 10^−2^	9.85 × 10^−1^
IFNA8	1.40	2.64	0.25	2.55	2.10 × 10^−2^	9.85 × 10^−1^
IL33	−1.07	0.47	−1.97	−0.18	2.25 × 10^−2^	9.85 × 10^−1^
CD274	−0.98	0.51	−1.82	−0.14	2.62 × 10^−2^	9.85 × 10^−1^
TNFSF13	−0.83	0.56	−1.55	−0.11	2.65 × 10^−2^	9.85 × 10^−1^
TPSAB1	−1.12	0.46	−2.17	−0.07	3.81 × 10^−2^	9.85 × 10^−1^
Peripheral blood samples						
CXCR6	−0.26	0.84	−0.43	−0.08	7.35 × 10^−3^	9.43 × 10^−1^
CLEC4C	−0.25	0.84	−0.44	−0.06	1.12 × 10^−2^	9.43 × 10^−1^
LY9	0.17	1.12	0.04	0.30	1.33 × 10^−2^	9.43 × 10^−1^
ITK	0.17	1.12	0.03	0.30	1.78 × 10^−2^	9.43 × 10^−1^
PNMA1	−0.16	0.90	−0.29	−0.03	1.89 × 10^−2^	9.43 × 10^−1^
CSF1	−0.25	0.84	−0.46	−0.04	2.11 × 10^−2^	9.43 × 10^−1^
FAS	−0.23	0.85	−0.42	−0.04	2.13 × 10^−2^	9.43 × 10^−1^
PVR	−0.19	0.88	−0.36	−0.03	2.54 × 10^−2^	9.43 × 10^−1^
AKT3	0.12	1.09	0.01	0.23	3.34 × 10^−2^	9.43 × 10^−1^
CD59	−0.19	0.88	−0.36	−0.01	3.51 × 10^−2^	9.43 × 10^−1^
AZE Group						
Nasal mucosa samples						
APOE	−1.68	0.31	−2.93	−0.43	1.19 × 10^−2^	9.90 × 10^−1^
TPTE	1.00	2.00	0.20	1.81	1.84 × 10^−2^	9.90 × 10^−1^
CAMP	1.19	2.28	0.12	2.26	3.15 × 10^−2^	9.90 × 10^−1^
CD27	0.59	1.51	0.03	1.16	3.94 × 10^−2^	9.90 × 10^−1^
IL23A	0.59	1.50	0.03	1.15	4.02 × 10^−2^	9.90 × 10^−1^
IL18RAP	1.13	2.19	−0.03	2.30	5.56 × 10^−2^	9.90 × 10^−1^
C4B	0.46	1.37	−0.04	0.95	6.71 × 10^−2^	9.90 × 10^−1^
CCR2	0.60	1.52	−0.08	1.28	7.84 × 10^−2^	9.90 × 10^−1^
CT45A1	0.70	1.62	−0.09	1.49	7.86 × 10^−2^	9.90 × 10^−1^
IL18	−0.43	0.74	−0.92	0.06	7.99 × 10^−2^	9.90 × 10^−1^
Peripheral blood samples						
NT5E	0.39	1.31	0.15	0.62	2.66 × 10^−3^	9.54 × 10^−1^
SPN	0.40	1.32	0.11	0.69	1.02 × 10^−2^	9.54 × 10^−1^
ITGAL	0.17	1.13	0.04	0.30	1.07 × 10^−2^	9.54 × 10^−1^
CCR4	−0.35	0.78	−0.62	−0.08	1.34 × 10^−2^	9.54 × 10^−1^
IL2RB	0.25	1.19	0.05	0.45	1.53 × 10^−2^	9.54 × 10^−1^
CEACAM8	−0.44	0.74	−0.79	−0.09	1.60 ×10^−2^	9.54 × 10^−1^
FYN	0.13	1.09	0.03	0.23	1.68 × 10^−2^	9.54 × 10^−1^
OAS3	0.30	1.23	0.05	0.55	1.97 × 10^−2^	9.54 × 10^−1^
SLC11A1	−0.22	0.86	−0.40	−0.03	2.50 × 10^−2^	9.54 × 10^−1^
IL6ST	0.16	1.12	0.02	0.30	2.59 × 10^−2^	9.54 × 10^−1^

FP had the greatest effect on immune gene expression, with 206 immune genes differentially expressed at *p* < .05 following treatment. Of these 206 differentially expressed genes (DEGs), 24 immune genes were upregulated (0.49–1.40 Log2 FC), and 182 immune genes were downregulated (−2.57 to −0.45 Log2 FC). For AZE/FP, a total of 16 immune genes were differentially expressed following treatment (*p* < .05); 10 genes were downregulated (−1.53 to −0.58 Log2 FC) and six genes were upregulated (1.07–1.62 Log2 FC). AZE had the least effect on immune gene expression; five immune genes were differentially expressed (*p* < .05) following treatment; one gene was downregulated (−1.68 Log2 FC) and four immune genes were upregulated (0.59 to 1.19 Log2 FC).

#### 3.3.2 Blood

Blood samples from 13 FP, 16 AZE/FP, and 15 AZE participants were available for analysis (Table S4). The number of DEGs from blood samples was lower than that observed in nasal lysate samples with 485 genes from the NanoString nCounter panel expressed above background. The top 20 differentially expressed genes for each treatment group are shown in Table 2. Treatment with FP had the greatest effect on immune gene expression, with 34 genes differentially expressed (*p* < .05); six genes were downregulated (−0.47 to −0.15 Log2 FC) and 24 genes were upregulated (0.16–0.50 Log2 FC). For AZE/FP, a total of 18 genes were differentially expressed (*p* < .05) following treatment; nine genes were downregulated (−0.26 to −0.15 Log2 FC) and nine were upregulated (0.12–0.20 Log2 FC). For AZE, a total of 20 genes were differentially expressed (*p* < .05); six genes were downregulated (−0.44 to −0.15 Log2 FC) and 14 genes were upregulated (0.13–0.43 Log2 FC). Given the modest impacts of treatment on differential gene expression in blood samples, additional analyses were not performed.

### 3.4 Comparison of immune gene expression between treatment groups

Four DEGs were in common between AZE/FP and FP; *TPSAB1, NOS2, CD274*, and *TNFSF13* all which were downregulated in both treatment groups (Table 3). There were no DEGs in common between AZE/FP and AZE, or between FP and AZE. For those DEGs identified for each of the treatments, the gene expression fold change was compared with the other treatment groups. The difference in fold change values between FP and AZE was the greatest, with 126 genes significantly different between these groups at *p* < .05 (Table S5). When comparing FP and AZE/FP, a total of 112 genes had significantly different FC values (Table S5). AZE/FP and AZE were the most similar when comparing FC values, with only eight genes significantly different between groups (ST 5).

**Table 3:** Differentially expressed genes (*p* < .05) in common between treatment group

	FP		AZE/FP		AZE		Between groups
	FC	*p* value	FC	*p* value	FC	*p* value	*p* value
TPSAB1	−2.04 (1.69)	<.0001	−1.12 (1.69)	.038	0.03 (1.75)	.956	FP and AZE *p* = .008
NOS2	−0.87 (1.21)	.018	−0.99 (1.56)	.048	0.25 (1.30)	.517	AZE/FP and AZE *p* = .057
							FP and AZE *p* = .04
CD274	−0.87 (1.57)	.049	−0.98 (1.29)	.026	0.37 (1.99)	.503	n/a
TNFSF13	−0.70 (0.90)	.042	−0.83 (1.04)	.026	0.18 (0.62)	.449	AZE/FP and AZE *p* = .012
							FP and AZE *p* = .012

*Note*: Data are presented as average fold change [FC] (standard deviation [*SD*]).

Abbreviations: AZE/FP, azelastine/fluticasone propionate “Dymista ®” group; FP, fluticasone propionate “Flixonase ®” group.

### 3.5 Pathway enrichment

The DEGs in the FP group were significantly enriched into 186 Reactome Pathways. The top four Reactome Pathways include Immune System, Cytokine Signaling in Immune system, Signaling by Interleukins, and Innate Immune System (Table 4). The DEGs in the AZE/FP group were significantly enriched into four Reactome Pathways (Table 4) including Hemostasis, Immune System, Cytokine Signaling in Immune System, and PI5P, PP2A, and IER3 Regulate PI3K/AKT Signaling. These four enriched pathways were also significantly enriched in the FP group (data not shown). The DEGs in the AZE group were not enriched into any Reactome Pathways.

**Table 4:** Top four Reactome pathways for FP and AZE/FP

Pathway	Description	Count in gene set	False discovery rate
FP			
HSA‐168256	Immune system	144 of 1925	1.02 × 10^−91^
HSA‐1280215	Cytokine signaling in immune system	79 of 654	3.69 × 10^−57^
HSA‐449147	Signaling by interleukins	62 of 439	2.63 × 10^−47^
HSA‐449147	Innate immune system	69 of 1012	3.14 × 10^−34^
AZE/FP			
HSA‐109582	Hemostasis	5 of 601	0.0059
HSA‐168256	Immune System	7 of 1925	0.0129
HSA‐1280215	Cytokine Signaling in Immune system	4 of 654	0.0389
HSA‐6811558	PI5P, PP2A, and IER3 Regulate PI3K/AKT Signaling	2 of 85	0.0449

*Note*: The differentially expressed genes from the AZE group were not enriched into any Reactome pathways.

Abbreviations: AZE/FP, azelastine/fluticasone propionate “Dymista®” group; FP, fluticasone propionate “Flixonase®” group.

## 4 DISCUSSION

The combination AZE/FP nasal spray has been reported to be significantly more effective at reducing self‐reported AR symptoms than treatment with either agent alone.^7^, ^18^ The biological basis for this enhanced AR symptom relief is unknown. The current study used a parallel‐group design to compare the immune gene expression profiles of nasal lysate samples from AR sufferers following administration with AZE/FP formulated together in comparison to products formulated with a single active ingredient (either AZE or FP alone) as control groups. AR symptoms significantly improved under all treatments, but the improvement was less pronounced with AZE.

A key finding in this study was distinct gene expression patterns between groups in response to treatment. FP had a strong downregulatory effect on gene expression, whilst effects of AZE/FP and AZE treatment were more modest with most DEGs being upregulated. A total of 206 DEGs were identified in the FP group. The majority of these were downregulated; consistent with the primary mechanism of action of corticosteroids.^19^ The top three downregulated DEGs were *AMICA1*, *GZMB*, and *LTB. AMICA1*, is involved in leukocyte migration and antigen processing and presentation pathways^20^ and its downregulation supports the potential for FP to modulate the early stages of the allergic response. Indeed, the high‐affinity IgE receptor (*FCER1A* gene), important for mast cell sensitization, was also significantly downregulated by FP treatment. Downregulation of *GZMB* by corticosteroids has been previously reported.^21^, ^22^ Granzyme B is a serine protease encoded by the *GZMB* gene, is expressed by a range of immune cells including mast cells^23^, ^24^ and is also involved in extracellular matrix proteolysis and cytokine processing.^25^, ^26^, ^27^ The *LTB* gene encodes lymphotoxin beta which is a member of the TNF cytokine family. Binding of lymphotoxin β to the LTβ receptor induces activation of transcription factor NF‐κB which is involved in the expression of many pro‐inflammatory molecules pertinent to the allergic response.^28^, ^29^ Hence, downregulation of this gene could result in moderation of the inflammation associated with the allergic response.

AZE had only modest effects on gene expression compared with the other treatments with only five genes differentially expressed. This finding is consistent with the known mechanism of antihistamines, whereby antihistamines interact specifically with histamine receptors, rather than exhibiting broad immune‐modulatory action. The top three DEGs following treatment with AZE were *APOE*, *TPTE*, and *CAMP*. Apolipoprotein E (APOE) is involved in the capture and delivery of lipid antigens to antigen‐presenting cells.^30^ Downregulation of *APOE* may provide symptomatic relief through preventing enhanced antigen presentation and downstream allergic inflammation. The *TPTE* gene is involved in signal transduction pathways, however, its specific role in allergic disease is not known. The *CAMP* gene encodes the cathelicidin‐related antimicrobial peptides and was upregulated following treatment with AZE. Cathelicidin has antimicrobial and immunoregulatory functions.^31^, ^32^ Reduced levels of cathelicidin were observed in the nasal lavage fluid of children suffering AR compared with controls,^33^ indicating that cathelicidin may be involved in the pathogenesis of AR.

Of the 16 DEGs in response to AZE/FP, six were upregulated and 10 downregulated. The top three downregulated DEGs included *TNFSF10*, *NOS2A*, and *PPBP*. *TNFSF10* induces the activation of transcription factor NF‐κB a known driver of inflammatory pathways pertinent to the allergic response. A *TNFSF10* knock out mouse‐model has been found to have reduced airway hyperactivity, peribronchial eosinophilia, and levels of mast cells in the airways compared with wild‐type mice.^34^ As such, downregulation of this gene could contribute to the improvement of AR symptoms. *NOS2A* encodes a nitric oxide synthase with the increased expression of *NOS2A* identified in bronchial biopsy samples from allergic asthmatics compared with healthy controls downregulated following use of inhaled corticosteroids.^35^ Pro‐Platelet Basic Protein (PPBP) is a powerful chemoattractant and activator for neutrophils and has been previously reported to be downregulated by glucocorticoids.^36^ Neutrophils contribute to allergic inflammation via the release of reactive oxygen species and proteases which damage the nasal epithelium and promote migration of effector cells.^37^, ^38^ Reduced neutrophil infiltration in the nasal mucosa as a result of reduced *PPBP* expression would likely confer reductions in AR symptoms.

The DEGs in response to FP and AZE/FP were found to be enriched into Reactome pathways. As was expected based simply on total number of DEGs, the DEGs in the FP group were enriched into a greater number of pathways than the DEGs in the AZE/FP. In contrast, the DEGs in the AZE group were not enriched into any Reactome pathways, given the limited numbers of genes involved. This result confirms that AZE acts on a small number of distinct genes to exert its clinical effects rather than modulating many genes within a single, or multiple concurrent pathways. In addition to the identification of some shared Reactome pathways, genes that were common between treatments were also explored. It was surprising to note that only four DEGs (*TPSAB1*, *NOS2*, *CD274*, and *TNFSF1*3) were shared between FP and AZE/FP. All four genes were downregulated and it was noted that the FC values for *NOS2*, *CD274*, and *TNFSF13* were lower in the AZE/FP group compared with FP or AZE alone, indicating possible synergistic effects. Furthermore, some 112 DEGs were expressed significantly differently between AZE/FP and FP confirming unique and not simply additive effects on immune regulatory pathways between these treatments. In contrast, AZE/FP and AZE were more similar when comparing the fold change values of DEG with only eight genes significantly different between groups. The difference in modulation of these eight genes between AZE/FP and AZE may explain the enhanced clinical effects of AZE/FP compared to AZE. Finally, the lack of shared DEGs between the FP and AZE group was anticipated given the known distinct mechanisms of action of these two drugs. Indeed, among the DEGs, 126 genes were significantly differentially regulated between FP and AZE.

In comparison to nasal lysate samples, there were limited effects of FP, AZE/FP, and AZE on gene expression in peripheral blood samples. Both numbers of DEGs and FC values were attenuated in peripheral blood. The translation of these small changes in gene expression to substantial protein production and meaningful clinical effects is generally considered unlikely. Indeed, second generation steroids such as FP have an estimated systemic bioavailability of less than 1% and systemic adverse events are considered rare.^39^ Intranasal application of a single dose of FP and Rhinocort (Budesonide) had no significant effect on peripheral blood lymphocyte populations of healthy individuals.^40^ Similarly, the small gene expression changes in blood samples following nasal spray administration reported here, maybe be indicative of some carry‐over effects from the topical site, but suggest that broad systemic effects as a result of topical administration are unlikely.

AZE/FP and FP had comparable effects on symptom relief. However, it is acknowledged that this study was not powered to detect statistically significant differences in clinical outcome between treatments. Similarly, if the documented symptomatic relief exceeded natural day‐to‐day variation in self‐reported symptoms is unclear in the absence of a placebo control. Regardless, the observations of greater reductions in self‐reported symptoms in response to FP and AZE/FP when compared to AZE alone are consistent with other published studies.^18^ Despite the comparable effects of AZE/FP and FP alone on symptom relief, effects on immune‐gene expression were not similar. Of particular interest, the combination AZE/FP spray, whilst achieving comparable symptom reduction to FP, did not induce greater changes in gene expression than either treatment alone, that is simple additive effects on gene expression were not observed. The moderate degree of modulation of genes by AZE/FP suggests than in combination, alternative pathways may underpin the observed reduction in symptoms, while mitigating a broader local immunosuppressive effect.^41^, ^42^

The current study was unique in its assessment of patterns of immune gene expression in response to pharmaceutical treatment at the site of application, the nasal mucosa, in individuals who were well characterized for allergic disease. Furthermore, by comparing the combination product to the single active ingredient formulations in our parallel‐group design, we were able to directly assess different gene expression patterns between treatments. It is however recognized that many of the DEGs identified in this study did not reach statistical significance when adjusted for false discovery rate and as such additional studies are needed to confirm the validity of the DEGs including with the further addition of a placebo control. In addition, the effect of the intranasal sprays on immune gene expression was evaluated at a single time point only (day 7); this limitation could be overcome and greater resolution of biological pathways may be achieved with serial sampling over the duration of the intervention. As this was a community‐based study, the comparative effect of each nasal sprays on early‐phase and late‐phase allergic responses is not known and should be studied under conditions of controlled allergen exposure.

In conclusion, investigation of nasal mucosa samples of AR sufferers following the intranasal application of AZE/FP, compared to FP and AZE alone, revealed distinct gene expression patterns across treatments. The greatest distinction between gene expression profiles was between FP and AZE which is indicative of the different mechanisms of action between corticosteroids and antihistamines. A compelling finding of this study was that FP and AZE/FP both had moderate effects on symptom reduction, but had diverse effects on gene expression. FP had a strong downregulatory effect on gene expression compared with AZE/FP which had an intermediate effect on gene expression with a mix of downregulated and upregulated genes following treatment. The moderate number of genes modulated by AZE/FP appears to be sufficient to significantly reduce AR symptoms, whilst avoiding total suppression of the local mucosal immune system.

## REGISTRATION

Australian and New Zealand Clinical Trial Registry (#ACTRN12616001439437).

## CONFLICT OF INTERESTS

The authors declare that there are no conflict of interests.

## AUTHOR CONTRIBUTIONS

Design: Annabelle M. Watts, Nicholas P. West, Amanda J. Cox, and Peter K. Smith. Data collection; Annabelle M. Wattsand Amanda J. Cox; Experiments: Annabelle M. Watts. Data analysis: Annabelle M. Watts, Ping Zhang, and Nicholas P. West. Drafting of manuscript: Annabelle M. Watts and Amanda J. Cox. Manuscript revision and approval: all authors

## Supporting information

Supporting information.

## Data Availability

The data that support the findings of this study are available from the corresponding author upon reasonable request.
