# Supplementary material for: Nasal immune gene expression in response to azelastine and fluticasone propionate combination or monotherapy
Source: Immun Inflamm Dis. 2021 Nov 23;10(3):e571. doi: 10.1002/iid3.571 (PMC8926499; doi:10.1002/iid3.571)
Supplement: Supplementary file 1 — Supporting information. [file IID3-10-e571-s001.docx]

**SUPPLEMENTARY MATERIAL**

**Supplementary Table 1**

Adverse events by treatment group assessed on the per-protocol population.

|  | **FP** | **AZE/FP** | **AZE** |
| --- | --- | --- | --- |
| **Event** |  |  |  |
| Sneezing after application | 2 | 0 | 2 |
| Itchy eyes | 2 | 0 | 0 |
| Bad scent | 2 | 0 | 0 |
| Runny nose after application | 1 | 0 | 1 |
| Itchy nose | 1 | 0 | 1 |
| Itchy eyes | 2 | 0 | 0 |
| Wheezing after application | 1 | 0 | 0 |
| Eye twitch | 1 | 0 | 0 |
| Bad/bitter/metallic/copper/funny taste | 1 | 11 | 3 |
| Dryness inside the nose | 0 | 1 | 0 |
| Feeling jittery | 0 | 1 | 0 |
| Worsened nasal congestion | 0 | 2 | 0 |
| Sinus pain | 0 | 1 | 0 |
| Soreness of nasal passages | 0 | 1 | 2 |

FP, fluticasone propionate ‘Flixonase ®’ Group; AZE/FP, azelastine hydrochloride/ fluticasone propionate ‘Dymista ®’ group; AZE, azelastine ‘Azep®’ group.

**Supplementary Table 2**

Symptom severity pre- and post- nasal spray application (per-protocol population).

|  | **Symptom severity** | **Day 0** | **Day 7** | **P value** |
| --- | --- | --- | --- | --- |
| FP  (n=14) | Mini rhinoconjunctivitis quality of life score  (0-6 U) | 2.90 ± 1.25 | 1.13 ± 1.05 | <0.001 |
|  | Total Nasal Symptom Score (0-12 U) | 5.93 ± 3.95 | 1.64 ± 2.34 | 0.004 |
|  | Other Allergic Rhinitis Symptoms (0-12 U) | 4.51 ± 3.89 | 1.14 ± 2.07 | 0.006 |
|  | Visual Analogue Scale (0-100 mm) | 54.18 ± 33.51 | 20.96 ± 25.30 | 0.003 |
|  | Total Ocular Symptom Score (0-9 U) | 3.57 ± 2.44 | 1.64 ± 2.10 | 0.025 |
| AZE/FP (n=16) | Mini rhinoconjunctivitis quality of life score  (0-6 U) | 2.66 ± 0.79 | 0.83 ± 0.50 | <0.001 |
|  | Total Nasal Symptom Score (0-12 U) | 4.00 ± 1.86 | 1.21 ± 1.42 | <0.001 |
|  | Other Allergic Rhinitis Symptoms (0-12 U) | 3.07 ± 2.16 | 0.81 ± 0.83 | <0.001 |
|  | Visual Analogue Scale (0-100 mm) | 48.16 ± 22.31 | 16.59 ± 20.42 | <0.001 |
|  | Total Ocular Symptom Score (0-9 U) | 2.00 ± 2.00 | 0.69 ± 0.87 | 0.009 |
| AZE  (n=16) | Mini rhinoconjunctivitis quality of life score  (0-6 U) | 3.00 ± 1.12 | 1.80 ± 1.29 | 0.001 |
|  | Total Nasal Symptom Score (0-12 U) | 7.06 ± 3.64 | 4.25 ± 3.42 | 0.008 |
|  | Other Allergic Rhinitis Symptoms (0-12 U) | 5.21 ± 3.47 | 3.06 ± 3.21 | 0.009 |
|  | Visual Analogue Scale (0-100 mm) | 60.50 ± 30.10 | 41.03 ± 29.52 | 0.010 |
|  | Total Ocular Symptom Score (0-9 U) | 3.19 ± 2.74 | 1.88 ± 2.09 | 0.011 |

Data is presented as mean ± standard deviation. n, number; U, unit, FP, fluticasone propionate ‘Flixonase ®’ group; AZE/FP, azelastine / fluticasone propionate ‘Dymista ®’ group; AZE, azelastine ‘Azep®’ group.

**Supplementary Table 3**

Baseline demographic and clinical measures (nasal lysate cohort)

|  | **FP** | **AZE/FP** | **AZE** | **P value** |
| --- | --- | --- | --- | --- |
| n | 13 | 11 | 11 | - |
| Age (years) | 39.11 ± 14.08 | 40.68 ± 11.34 | 31.97 ± 11.82 | 0.234 |
| Sex F/M (% Female) | 9/4 (69%) | 6/5 (55%) | 8/3 (72%) | 0.631 |
| Height (cm) | 169.04 ± 9.73 | 171.68 ± 9.46 | 171.59 ± 8.29 | 0.725 |
| Weight (kg) | 77.21 ± 16.24 | 75.15 ± 14.32 | 73.14 ± 13.65 | 0.801 |
| BMI (kg/m^2^) | 26.83 ± 4.05 | 25.34 ± 3.38 | 24.80 ± 4.12 | 0.417 |
| Ethnicity (% Caucasian) | 76.92% | 55% | 100% | 0.040 |
| **Immune measures** |  |  |  |  |
| White cell count (x10^9^/L) | 7.32 ± 1.67 | 6.74 ± 1.40 | 5.96 ± 1.69 | 0.132 |
| Lymphocytes (x10^9^/L) | 2.31 ± 0.83 | 2.09 ± 0.51 | 2.06 ± 0.71 | 0.647 |
| Eosinophils (x10^9^/L) | 0.55 ± 0.38 | 0.43 ± 0.28 | 0.26 ± 0.17 | 0.044 |
| Neutrophils (x10^9^/L) | 3.87 ± 1.17 | 3.62 ± 1.00 | 3.15 ± 1.11 | 0.285 |
| Basophils (x10^9^/L) | 0.06 ± 0.05 | 0.07 ± 0.02 | 0.04 ± 0.04 | 0.278 |
| ESR (mm/hr) | 14.15 ± 14.28 | 9.09 ± 7.08 | 6.27 ± 6.93 | 0.102 |
| **Allergen sensitivity** |  |  |  |  |
| Co-allergy to dust mites and pollen (%) | 53.8% | 54.5% | 54.5% | 1.00 |
| IgE *D. pteronyssinus* (kU/L) | 38.74 ± 41.98 | 8.26 ± 8.66 | 15.79 ± 23.46 | 0.055 |
| IgE *D. farinae* (kU/L) | 34.82 ± 41.47 | 5.67 ± 5.76 | 11.89 ± 20.24 | 0.059 |
| IgE grass pollen mix (kU/L) | 2.29 ± 4.43 | 10.37 ± 29.99 | 8.45 ± 25.71 | 0.648 |
| IgG4 *D. pteronyssinus* (kU/L) | 0.49 ± 0.57 | 0.46 ± 0.49 | 0.46 ± 0.48 | 0.988 |
| IgG4 *D. farinae* (kU/L) | 0.40 ± 0.43 | 0.38 ± 0.28 | 0.36 ± 0.42 | 0.964 |
| IgG4 grass pollen mix (kU/L) | 0.64 ± 0.35 | 0.78 ± 0.57 | 0.89 ± 0.72 | 0.946 |
| **Symptom severity (day 0)** |  |  |  |  |
| Total Nasal Symptom Score (0-12 U) | 6.15 ± 4.02 | 3.82 ± 1.83 | 6.45 ± 3.78 | 0.064 |
| Total Ocular Symptom Score (0-9 U) | 3.85 ± 2.30 | 1.82 ± 2.09 | 2.18 ± 2.60 | 0.089 |
| Mini rhinoconjunctivitis quality of life score (0-6 U) | 2.90 ± 1.30 | 2.60 ± 0.73 | 2.73 ± 1.24 | 0.811 |
| Other Allergic Rhinitis Symptoms (0-12 U) | 4.78 ± 3.90 | 3.02 ± 2.21 | 4.40 ± 3.43 | 0.411 |
| Visual Analogue Scale (0-100 mm) | 56.88 ± 33.25 | 46.23 ± 22.54 | 59.64 ± 32.04 | 0.542 |
| **Medication Usage** |  |  |  |  |
| Allergy medication use; % of total diary responses (washout period) | 0.38 ± 0.30 | 0.22 ± 0.19 | 0.36 ± 0.31 | 0.351 |

n, number; L, Litre; kU, Kilounit; U, unit; mm, millimetre; %, percentage; FP, fluticasone propionate ‘Flixonase ®’ Group; AZE/FP, azelastine / fluticasone propionate ‘Dymista ®’ group; AZE, azelastine ‘Azep®’ group.

**Supplementary Table 4**

Baseline demographic and clinical measures (peripheral blood samples)

|  | **FP** | **AZE/FP** | **AZE** | **P value** |
| --- | --- | --- | --- | --- |
| n | 13 | 16 | 15 | - |
| Age (years) | 38.47 ± 14.85 | 39.43 ± 10.03 | 35.67 ± 14.16 | 0.712 |
| Sex F/M (% Female) | 9/4 (69%) | 10/6 (~63%) | 10/5 (67%) | 0.928 |
| Height (cm) | 169.85 ± 9.30 | 171.03 ± 10.26 | 172.27 ± 8.83 | 0.799 |
| Weight (kg) | 76.91 ± 16.34 | 72.16 ± 15.33 | 73.03 ± 13.55 | 0.679 |
| BMI (kg/m^2^) | 26.46 ± 4.05 | 24.45 ± 3.37 | 24.52 ± 3.62 | 0.275 |
| Ethnicity (% Caucasian) | 76.92% | 68.75% | 86.67% | 0.493 |
| **Immune measures** |  |  |  |  |
| White cell count (x10^9^/L) | 7.05 ± 1.69 | 6.98 ± 2.09 | 5.80 ± 1.50 | 0.116 |
| Lymphocytes (x10^9^/L) | 2.31 ± 0.83 | 2.26 ± 0.73 | 1.97 ± 0.65 | 0.403 |
| Eosinophils (x10^9^/L) | 0.55 ± 0.38 | 0.44 ± 0.30 | 0.26 ± 0.17 | 0.033 |
| Neutrophils (x10^9^/L) | 3.59 ± 1.13 | 3.68 ± 1.33 | 3.10 ± 0.98 | 0.349 |
| Basophils (x10^9^/L) | 0.07 ± 0.05 | 0.07 ± 0.03 | 0.04 ± 0.03 | 0.045 |
| ESR (mm/hr) | 12.85 ± 12.82 | 7.94 ± 6.17 | 7.07 ± 7.77 | 0.383 |
| **Allergen sensitivity** |  |  |  |  |
| Co-allergy to dust mites and pollen (%) | 53.8% | 62.5% | 60% | 0.928 |
| IgE *D. pteronyssinus* (kU/L) | 41.55 ± 41.10 | 17.12 ± 21.60 | 11.82 ± 20.97 | 0.087 |
| IgE *D. farinae* (kU/L) | 37.30 ± 40.60 | 13.41 ± 18.31 | 8.90 ± 17.87 | 0.091 |
| IgE grass pollen mix (kU/L) | 2.29 ± 4.42 | 10.44 ± 25.84 | 6.56 ± 21.99 | 0.567 |
| IgG4 *D. pteronyssinus* (kU/L) | 0.52 ± 0.56 | 0.47 ± 0.42 | 0.38 ± 0.43 | 0.735 |
| IgG4 *D. farinae* (kU/L) | 0.43 ± 0.43 | 0.39 ± 0.26 | 0.30 ± 0.38 | 0.581 |
| IgG4 grass pollen mix (kU/L) | 0.66 ± 0.36 | 1.07 ± 0.97 | 0.80 ± 0.66 | 0.303 |
| **Symptom severity (day 0)** |  |  |  |  |
| Total Nasal Symptom Score (0-12 U) | 5.46 ± 3.69 | 4.00 ± 1.86 | 6.87 ± 3.68 | 0.301 |
| Total Ocular Symptom Score (0-9 U) | 3.38 ± 2.43 | 2.00 ± 2.00 | 3.00 ± 2.73 | 0.277 |
| Mini rhinoconjunctivitis quality of life score (0-6 U) | 2.85 ± 1.28 | 2.66 ± 0.78 | 2.95 ± 1.15 | 0.746 |
| Other Allergic Rhinitis Symptoms (0-12 U) | 4.32 ± 3.97 | 3.07 ± 2.16 | 5.03 ± 3.51 | 0.248 |
| Visual Analogue Scale (0-100 mm) | 51.19 ± 32.88 | 48.16 ± 22.31 | 58.80 ± 30.35 | 0.573 |
| **Medication Usage** |  |  |  |  |
| Allergy medication use; % of total diary responses (washout period) | 0.38 ± 0.30 | 0.25 ± 0.20 | 0.41 ± 0.32 | 0.221 |

n, number; L, Litre; kU, Kilounit; U, unit; mm, millimetre; %, percentage; FP, fluticasone propionate ‘Flixonase ®’ group; AZE/FP, azelastine / fluticasone propionate ‘Dymista ®’ group; AZE, azelastine ‘Azep®’ group.

**Supplementary Table 5**

Fold Change values between groups.

| **FP – AZE/FP** | | | | | |
| --- | --- | --- | --- | --- | --- |
|  | **log2 fold change** | **Lower confidence limit (Log2)** | **Upper confidence limit (log2)** | **P value** | **P adjust** |
| CEACAM1 | -1.50 | -2.33 | -0.66 | 0.001 | 0.161 |
| FLT3LG | -1.42 | -2.22 | -0.61 | 0.001 | 0.161 |
| PTGDR2 | -2.47 | -3.90 | -1.03 | 0.001 | 0.161 |
| FCER1A | -2.07 | -3.30 | -0.84 | 0.002 | 0.161 |
| NFATC2 | -1.59 | -2.55 | -0.63 | 0.002 | 0.161 |
| CD96 | -2.15 | -3.45 | -0.84 | 0.002 | 0.161 |
| SOCS1 | -2.02 | -3.27 | -0.76 | 0.002 | 0.161 |
| SPN | -2.02 | -3.29 | -0.75 | 0.003 | 0.161 |
| DUSP6 | -1.71 | -2.80 | -0.62 | 0.003 | 0.161 |
| LTB | -2.63 | -4.31 | -0.95 | 0.003 | 0.161 |
| FYN | -1.52 | -2.50 | -0.54 | 0.003 | 0.161 |
| IL1RL1 | -2.06 | -3.40 | -0.73 | 0.003 | 0.161 |
| CFD | -2.09 | -3.45 | -0.72 | 0.004 | 0.161 |
| IL4 | -1.87 | -3.11 | -0.64 | 0.004 | 0.161 |
| INPP5D | -1.82 | -3.03 | -0.62 | 0.004 | 0.161 |
| RUNX3 | -1.58 | -2.66 | -0.51 | 0.005 | 0.172 |
| TCF7 | -1.24 | -2.09 | -0.39 | 0.005 | 0.172 |
| ABCB1 | -1.78 | -3.00 | -0.56 | 0.005 | 0.172 |
| PTGS1 | -1.95 | -3.29 | -0.61 | 0.006 | 0.172 |
| C2 | 1.13 | 0.35 | 1.91 | 0.006 | 0.172 |
| TARP | -2.31 | -3.94 | -0.69 | 0.007 | 0.184 |
| MST1R | 1.53 | 0.44 | 2.61 | 0.007 | 0.189 |
| IL2RA | -2.11 | -3.64 | -0.59 | 0.008 | 0.199 |
| TGFB1 | -1.81 | -3.12 | -0.50 | 0.008 | 0.200 |
| CD1A | -1.87 | -3.24 | -0.49 | 0.009 | 0.200 |
| CXCR6 | -1.59 | -2.77 | -0.42 | 0.009 | 0.200 |
| CD1D | -1.70 | -2.95 | -0.44 | 0.009 | 0.200 |
| CXCR3 | -1.53 | -2.66 | -0.40 | 0.010 | 0.200 |
| BTK | -1.96 | -3.41 | -0.50 | 0.010 | 0.201 |
| CARD9 | -1.25 | -2.19 | -0.31 | 0.011 | 0.201 |
| GZMB | -1.63 | -2.85 | -0.40 | 0.011 | 0.201 |
| PIK3CG | -1.70 | -3.01 | -0.40 | 0.012 | 0.205 |
| ITGA4 | -1.73 | -3.06 | -0.40 | 0.012 | 0.205 |
| AMICA1 | -2.33 | -4.13 | -0.53 | 0.013 | 0.205 |
| BCL2 | -1.32 | -2.35 | -0.29 | 0.013 | 0.205 |
| CYSLTR2 | -2.37 | -4.22 | -0.53 | 0.013 | 0.205 |
| CCL28 | 1.51 | 0.33 | 2.68 | 0.014 | 0.205 |
| SMPD3 | -1.36 | -2.43 | -0.29 | 0.014 | 0.205 |
| IFNA8 | -1.68 | -3.00 | -0.36 | 0.014 | 0.205 |
| MEF2C | -1.68 | -3.01 | -0.35 | 0.015 | 0.205 |
| CD79A | -2.10 | -3.76 | -0.43 | 0.015 | 0.205 |
| PAX5 | -1.63 | -2.94 | -0.33 | 0.016 | 0.205 |
| CD48 | -2.14 | -3.86 | -0.43 | 0.016 | 0.205 |
| ITK | -1.62 | -2.92 | -0.32 | 0.016 | 0.205 |
| CCR3 | -2.27 | -4.09 | -0.45 | 0.016 | 0.205 |
| FCGR2B | -2.03 | -3.66 | -0.40 | 0.016 | 0.205 |
| CCL23 | -2.02 | -3.67 | -0.38 | 0.017 | 0.207 |
| IRF4 | -1.66 | -3.00 | -0.31 | 0.018 | 0.207 |
| CCND3 | -0.92 | -1.68 | -0.17 | 0.018 | 0.207 |
| CD4 | -1.67 | -3.03 | -0.30 | 0.018 | 0.207 |
| CCR6 | -1.27 | -2.31 | -0.23 | 0.018 | 0.207 |
| IL2RB | -1.42 | -2.58 | -0.25 | 0.019 | 0.207 |
| CD1C | -1.57 | -2.87 | -0.27 | 0.019 | 0.207 |
| MS4A1 | -2.19 | -4.00 | -0.38 | 0.019 | 0.207 |
| CD84 | -1.60 | -2.93 | -0.27 | 0.020 | 0.209 |
| ITGB4 | 0.97 | 0.15 | 1.79 | 0.021 | 0.221 |
| ENTPD1 | -1.41 | -2.61 | -0.22 | 0.022 | 0.225 |
| ETS1 | -1.22 | -2.26 | -0.18 | 0.023 | 0.229 |
| TYK2 | -0.85 | -1.57 | -0.12 | 0.023 | 0.229 |
| LILRB2 | -2.08 | -3.85 | -0.30 | 0.023 | 0.229 |
| CFP | -1.83 | -3.41 | -0.25 | 0.025 | 0.229 |
| CD207 | -1.69 | -3.15 | -0.22 | 0.025 | 0.229 |
| IL32 | -1.63 | -3.04 | -0.21 | 0.025 | 0.229 |
| HLA-DOB | -1.26 | -2.35 | -0.16 | 0.026 | 0.229 |
| ITGAL | -2.02 | -3.78 | -0.26 | 0.026 | 0.229 |
| CD79B | -1.46 | -2.73 | -0.18 | 0.026 | 0.229 |
| IL10RA | -1.78 | -3.35 | -0.22 | 0.027 | 0.229 |
| ALOX 5 | -1.77 | -3.33 | -0.22 | 0.027 | 0.229 |
| RNASE3 | -1.35 | -2.53 | -0.16 | 0.027 | 0.229 |
| CSF2RB | -2.24 | -4.21 | -0.27 | 0.027 | 0.229 |
| LILRA1 | -1.83 | -3.47 | -0.18 | 0.030 | 0.248 |
| IL12RB1 | -1.46 | -2.77 | -0.15 | 0.030 | 0.248 |
| IL2RG | -1.83 | -3.49 | -0.18 | 0.031 | 0.250 |
| NCF4 | -1.96 | -3.74 | -0.19 | 0.031 | 0.250 |
| NLRC5 | -0.91 | -1.75 | -0.08 | 0.033 | 0.255 |
| CD27 | -1.60 | -3.06 | -0.13 | 0.033 | 0.255 |
| HRAS | 0.96 | 0.08 | 1.84 | 0.034 | 0.255 |
| CKLF | -1.49 | -2.86 | -0.11 | 0.035 | 0.255 |
| CYSLTR1 | -1.53 | -2.94 | -0.12 | 0.035 | 0.255 |
| TFEB | -0.95 | -1.83 | -0.07 | 0.035 | 0.255 |
| HLA-DRA | -1.19 | -2.30 | -0.08 | 0.036 | 0.255 |
| APP | 0.94 | 0.06 | 1.82 | 0.037 | 0.255 |
| LAIR2 | -1.63 | -3.15 | -0.10 | 0.037 | 0.255 |
| EGR2 | -1.84 | -3.57 | -0.11 | 0.037 | 0.255 |
| FCGR1A | -1.65 | -3.21 | -0.09 | 0.038 | 0.255 |
| IL13 | -1.18 | -2.29 | -0.06 | 0.039 | 0.255 |
| BTLA | -1.34 | -2.60 | -0.07 | 0.039 | 0.255 |
| CASP8 | -1.10 | -2.14 | -0.06 | 0.039 | 0.255 |
| CYLD | -0.92 | -1.80 | -0.05 | 0.039 | 0.255 |
| SYK | -1.09 | -2.13 | -0.06 | 0.040 | 0.255 |
| IKBKE | 0.60 | 0.03 | 1.18 | 0.040 | 0.255 |
| RIPK2 | -1.24 | -2.42 | -0.06 | 0.040 | 0.255 |
| CCL17 | -1.40 | -2.73 | -0.06 | 0.040 | 0.255 |
| CREB5 | -1.67 | -3.26 | -0.07 | 0.041 | 0.256 |
| IL18R1 | -1.59 | -3.11 | -0.06 | 0.042 | 0.257 |
| ITGAM | -1.83 | -3.58 | -0.07 | 0.042 | 0.257 |
| CD244 | -1.86 | -3.65 | -0.07 | 0.042 | 0.257 |
| CD37 | -2.02 | -3.98 | -0.06 | 0.044 | 0.257 |
| TBX21 | -1.66 | -3.29 | -0.04 | 0.045 | 0.257 |
| PSEN2 | 0.59 | 0.01 | 1.17 | 0.045 | 0.257 |
| JAK2 | -0.68 | -1.35 | -0.02 | 0.045 | 0.257 |
| IL4R | -1.00 | -1.98 | -0.02 | 0.046 | 0.257 |
| PIK3CD | -1.61 | -3.18 | -0.03 | 0.046 | 0.257 |
| IL23A | -0.93 | -1.85 | -0.01 | 0.047 | 0.257 |
| ITGB2 | -1.62 | -3.21 | -0.02 | 0.047 | 0.257 |
| NOTCH1 | -1.16 | -2.31 | -0.01 | 0.048 | 0.257 |
| KIT | 1.10 | 0.01 | 2.20 | 0.048 | 0.257 |
| CX3CL1 | 1.06 | 0.01 | 2.11 | 0.048 | 0.257 |
| NOD1 | -0.73 | -1.46 | 0.00 | 0.049 | 0.257 |
| FUT7 | -1.76 | -3.51 | 0.00 | 0.050 | 0.257 |
| IL13RA1 | -0.66 | -1.32 | 0.00 | 0.050 | 0.257 |
| POU2F2 | -1.09 | -2.19 | 0.00 | 0.050 | 0.257 |

FP, fluticasone propionate ‘Flixonase ®’ group; AZE/FP, azelastine hydrochloride / fluticasone propionate ‘Dymista ®’ group.

| **FP - AZE** | | | | | |
| --- | --- | --- | --- | --- | --- |
|  | **Log2 fold change** | **Lower confidence limit (Log2)** | **Upper confidence limit (log2)** | **P value** | **P adjust** |
| GZMB | -2.53 | -3.75 | -1.30 | 0.000 | 0.090 |
| FCER1A | -2.41 | -3.64 | -1.18 | 0.000 | 0.090 |
| SOCS1 | -2.36 | -3.61 | -1.10 | 0.001 | 0.090 |
| PTGDR2 | -2.66 | -4.10 | -1.22 | 0.001 | 0.090 |
| RUNX3 | -1.90 | -2.98 | -0.83 | 0.001 | 0.115 |
| LTB | -2.80 | -4.48 | -1.12 | 0.002 | 0.115 |
| AMICA1 | -3.00 | -4.80 | -1.20 | 0.002 | 0.115 |
| PIK3CG | -2.15 | -3.46 | -0.85 | 0.002 | 0.115 |
| CEACAM1 | -1.35 | -2.19 | -0.51 | 0.002 | 0.115 |
| CCL23 | -2.65 | -4.30 | -1.00 | 0.002 | 0.115 |
| IL1RL1 | -2.13 | -3.46 | -0.80 | 0.003 | 0.115 |
| IRF4 | -2.14 | -3.49 | -0.80 | 0.003 | 0.115 |
| CD96 | -2.07 | -3.37 | -0.77 | 0.003 | 0.115 |
| IFI35 | -1.25 | -2.04 | -0.45 | 0.003 | 0.115 |
| NOD1 | -1.14 | -1.87 | -0.42 | 0.003 | 0.115 |
| DUSP6 | -1.66 | -2.75 | -0.57 | 0.004 | 0.118 |
| HLA-DPA1 | -1.93 | -3.20 | -0.66 | 0.004 | 0.118 |
| TNFSF12 | -1.81 | -3.00 | -0.62 | 0.004 | 0.118 |
| SMPD3 | -1.61 | -2.68 | -0.55 | 0.004 | 0.118 |
| FCGR2B | -2.44 | -4.06 | -0.81 | 0.004 | 0.118 |
| MS4A2 | -2.10 | -3.50 | -0.70 | 0.004 | 0.118 |
| TPSAB1 | -2.07 | -3.46 | -0.69 | 0.004 | 0.118 |
| ALCAM | 1.33 | 0.44 | 2.22 | 0.005 | 0.118 |
| SELPLG | -2.64 | -4.43 | -0.85 | 0.005 | 0.121 |
| HLA-DRA | -1.63 | -2.74 | -0.52 | 0.005 | 0.121 |
| PAX5 | -1.91 | -3.21 | -0.60 | 0.005 | 0.121 |
| CD1A | -2.00 | -3.38 | -0.63 | 0.006 | 0.121 |
| CSF2RB | -2.80 | -4.78 | -0.83 | 0.007 | 0.126 |
| TARP | -2.31 | -3.93 | -0.68 | 0.007 | 0.126 |
| CD74 | -1.50 | -2.57 | -0.44 | 0.007 | 0.126 |
| SPN | -1.79 | -3.06 | -0.51 | 0.007 | 0.126 |
| CXCR3 | -1.59 | -2.72 | -0.45 | 0.007 | 0.126 |
| CCR3 | -2.54 | -4.36 | -0.72 | 0.008 | 0.126 |
| CKLF | -1.91 | -3.29 | -0.54 | 0.008 | 0.126 |
| PAFAH2 | -1.09 | -1.88 | -0.31 | 0.008 | 0.126 |
| INPP5D | -1.67 | -2.88 | -0.47 | 0.008 | 0.126 |
| BTK | -2.02 | -3.48 | -0.56 | 0.008 | 0.126 |
| CFD | -1.88 | -3.25 | -0.52 | 0.008 | 0.126 |
| CYSLTR1 | -1.92 | -3.33 | -0.50 | 0.009 | 0.137 |
| IL16 | -2.32 | -4.03 | -0.61 | 0.009 | 0.137 |
| LGALS3 | 1.41 | 0.35 | 2.46 | 0.010 | 0.148 |
| CYSLTR2 | -2.44 | -4.28 | -0.59 | 0.011 | 0.154 |
| C2 | 1.02 | 0.24 | 1.81 | 0.012 | 0.155 |
| NCF4 | -2.33 | -4.10 | -0.55 | 0.012 | 0.155 |
| FLT3LG | -1.05 | -1.85 | -0.24 | 0.012 | 0.155 |
| CD1C | -1.68 | -2.97 | -0.38 | 0.013 | 0.155 |
| CASP8 | -1.35 | -2.39 | -0.31 | 0.013 | 0.155 |
| ICAM3 | -2.07 | -3.67 | -0.47 | 0.013 | 0.155 |
| PTGS1 | -1.73 | -3.07 | -0.39 | 0.013 | 0.155 |
| ITGA4 | -1.71 | -3.04 | -0.38 | 0.013 | 0.155 |
| SYK | -1.32 | -2.35 | -0.28 | 0.014 | 0.164 |
| MUC1 | 1.58 | 0.31 | 2.84 | 0.016 | 0.177 |
| ITGB2 | -1.96 | -3.55 | -0.36 | 0.018 | 0.177 |
| IL18 | 0.77 | 0.14 | 1.40 | 0.018 | 0.177 |
| FYN | -1.20 | -2.18 | -0.22 | 0.018 | 0.177 |
| ITGAM | -2.14 | -3.90 | -0.38 | 0.018 | 0.177 |
| CTLA4 | -2.09 | -3.82 | -0.37 | 0.018 | 0.177 |
| IFIT1 | -1.86 | -3.40 | -0.33 | 0.019 | 0.177 |
| CD207 | -1.77 | -3.24 | -0.31 | 0.019 | 0.177 |
| ISG15 | -1.33 | -2.43 | -0.23 | 0.019 | 0.177 |
| CLEC4C | -2.04 | -3.73 | -0.35 | 0.020 | 0.177 |
| CYLD | -1.05 | -1.93 | -0.18 | 0.020 | 0.177 |
| ITGB4 | 0.98 | 0.16 | 1.79 | 0.020 | 0.177 |
| ALOX 5 | -1.86 | -3.42 | -0.31 | 0.020 | 0.177 |
| JAK2 | -0.79 | -1.46 | -0.13 | 0.021 | 0.177 |
| TGFB1 | -1.56 | -2.87 | -0.25 | 0.021 | 0.177 |
| CCND3 | -0.90 | -1.65 | -0.14 | 0.021 | 0.177 |
| CLDN1 | 1.12 | 0.18 | 2.07 | 0.021 | 0.177 |
| PIK3CD | -1.87 | -3.44 | -0.29 | 0.021 | 0.177 |
| HLA-DRB3 | -1.51 | -2.79 | -0.24 | 0.022 | 0.177 |
| HLA-DMB | -1.06 | -1.96 | -0.16 | 0.022 | 0.177 |
| IL13 | -1.31 | -2.43 | -0.20 | 0.022 | 0.177 |
| PSMB9 | -1.12 | -2.06 | -0.17 | 0.022 | 0.177 |
| NFATC2 | -1.13 | -2.09 | -0.17 | 0.022 | 0.177 |
| CDH1 | 1.18 | 0.18 | 2.18 | 0.023 | 0.177 |
| TNFSF13 | -0.88 | -1.63 | -0.12 | 0.024 | 0.184 |
| TYK2 | -0.84 | -1.56 | -0.11 | 0.024 | 0.184 |
| APOE | 1.69 | 0.23 | 3.15 | 0.025 | 0.184 |
| PTPRC | -2.49 | -4.65 | -0.34 | 0.025 | 0.184 |
| IRF7 | -1.24 | -2.31 | -0.16 | 0.025 | 0.184 |
| HLA-DPB1 | -2.43 | -4.55 | -0.31 | 0.026 | 0.187 |
| CD79A | -1.90 | -3.57 | -0.23 | 0.027 | 0.190 |
| RNASE3 | -1.34 | -2.52 | -0.15 | 0.028 | 0.194 |
| CD163 | 1.50 | 0.17 | 2.82 | 0.028 | 0.194 |
| IL17RA | -1.27 | -2.40 | -0.14 | 0.029 | 0.194 |
| CCL13 | -1.63 | -3.08 | -0.18 | 0.029 | 0.194 |
| MEF2C | -1.49 | -2.82 | -0.16 | 0.029 | 0.194 |
| CD244 | -2.00 | -3.79 | -0.21 | 0.029 | 0.194 |
| IL32 | -1.58 | -3.00 | -0.17 | 0.030 | 0.194 |
| SELL | -2.16 | -4.11 | -0.22 | 0.030 | 0.194 |
| ITGAL | -1.96 | -3.73 | -0.20 | 0.030 | 0.194 |
| ITGB1 | 0.70 | 0.07 | 1.32 | 0.030 | 0.194 |
| OSM | -2.56 | -4.88 | -0.24 | 0.032 | 0.195 |
| RIPK2 | -1.30 | -2.48 | -0.12 | 0.032 | 0.195 |
| MS4A1 | -1.99 | -3.80 | -0.18 | 0.032 | 0.195 |
| LCP1 | -2.12 | -4.04 | -0.19 | 0.032 | 0.195 |
| EGR1 | -1.71 | -3.27 | -0.15 | 0.033 | 0.195 |
| LILRB2 | -1.95 | -3.73 | -0.17 | 0.033 | 0.195 |
| IL2RB | -1.28 | -2.44 | -0.11 | 0.033 | 0.195 |
| CFP | -1.72 | -3.30 | -0.14 | 0.033 | 0.197 |
| NFATC3 | -0.68 | -1.30 | -0.05 | 0.035 | 0.202 |
| IL3RA | -1.59 | -3.06 | -0.12 | 0.035 | 0.202 |
| TLR1 | -1.49 | -2.87 | -0.10 | 0.036 | 0.204 |
| IFIT2 | -1.62 | -3.13 | -0.11 | 0.037 | 0.205 |
| HCK | -2.05 | -3.96 | -0.13 | 0.037 | 0.205 |
| TNFRSF10C | -2.24 | -4.35 | -0.13 | 0.038 | 0.206 |
| CD37 | -2.08 | -4.04 | -0.12 | 0.038 | 0.206 |
| ENTPD1 | -1.27 | -2.46 | -0.07 | 0.039 | 0.206 |
| CD48 | -1.81 | -3.53 | -0.10 | 0.039 | 0.206 |
| IL23A | -0.97 | -1.88 | -0.05 | 0.039 | 0.206 |
| F13A1 | -1.56 | -3.03 | -0.08 | 0.039 | 0.206 |
| EGR2 | -1.82 | -3.55 | -0.09 | 0.040 | 0.206 |
| IL5RA | -1.05 | -2.06 | -0.05 | 0.040 | 0.206 |
| IL18R1 | -1.60 | -3.13 | -0.08 | 0.040 | 0.206 |
| CYBB | -2.17 | -4.25 | -0.09 | 0.042 | 0.212 |
| JAK3 | -1.69 | -3.31 | -0.06 | 0.042 | 0.212 |
| IL10RA | -1.63 | -3.19 | -0.06 | 0.042 | 0.212 |
| CD180 | -1.19 | -2.34 | -0.04 | 0.043 | 0.212 |
| IL4 | -1.27 | -2.50 | -0.04 | 0.044 | 0.215 |
| SMAD3 | 0.71 | 0.02 | 1.40 | 0.044 | 0.215 |
| CR1 | -1.96 | -3.89 | -0.03 | 0.047 | 0.226 |
| FUT7 | -1.78 | -3.53 | -0.02 | 0.047 | 0.226 |
| PNMA1 | 0.72 | 0.01 | 1.43 | 0.047 | 0.226 |
| PECAM1 | -1.94 | -3.87 | -0.02 | 0.048 | 0.227 |
| NOS2 | -1.12 | -2.23 | -0.01 | 0.048 | 0.227 |
| TAP1 | -0.83 | -1.65 | 0.00 | 0.049 | 0.229 |

FP, fluticasone propionate ‘Flixonase ®’ Group; AZE, azelastine hydrochloride ‘Azep®’ group.

| **AZE/FP - AZE** | | | | | |
| --- | --- | --- | --- | --- | --- |
|  | **Log2 fold change** | **Lower confidence limit (Log2)** | **Upper confidence limit (log2)** | **P value** | **P adjust** |
| TNFSF10 | -1.02 | -1.64 | -0.39 | 0.002 | 0.997 |
| NOS2A | -1.76 | -3.03 | -0.48 | 0.008 | 0.997 |
| TNFSF13 | -1.01 | -1.80 | -0.22 | 0.013 | 0.997 |
| APOE | 1.87 | 0.35 | 3.39 | 0.017 | 0.997 |
| IL18 | 0.77 | 0.11 | 1.42 | 0.023 | 0.997 |
| NOS2 | -1.24 | -2.40 | -0.08 | 0.037 | 0.997 |
| CD163 | 1.44 | 0.06 | 2.82 | 0.042 | 0.997 |
| MARCO | 1.47 | 0.04 | 2.89 | 0.044 | 0.997 |

AZE/FP, azelastine hydrochloride / fluticasone propionate ‘Dymista ®’ group; AZE, azelastine hydrochloride ‘Azep®’ group.
